# A Novel Study Quantifying Intrinsic Dimensional Variation Among Glaucoma Drainage Devices

**DOI:** 10.7759/cureus.17771

**Published:** 2021-09-06

**Authors:** Jesse A Terrell, Charles Richard Blake

**Affiliations:** 1 Ophthalmology, Baylor College of Medicine, Houston, USA; 2 Ophthalmology, University of Florida, Gainesville, USA

**Keywords:** glaucoma drainage device, glaucoma, quality improvement, molteno, baerveldt

## Abstract

Purpose

Variation among aqueous humor outflow from venting slits performed on glaucoma drainage device tubing often occurs even when physician technique and equipment are held constant. Our hypothesis is that there are dimensional differences within the tubing, even among the same make and model of glaucoma drainage device (GDD) implants.

Methods

Prior to surgical implantation, excess glaucoma drainage tubing was collected for analysis. The tubing samples were sliced horizontally, and the external tube, internal lumen, and wall dimension measurements were collected microscopically. Groups were divided based upon brand and model and then statistically analyzed using an independent t-test. A total of 28 tubes were analyzed, consisting of 7 Molteno and 21 Baerveldt implants.

Results

The mean external diameter for the Molteno group was 656 ± 20µm, significantly larger than the Baerveldt external diameter of 620 ± 13µm (P<0.05). The mean internal diameter among Molteno lumens was 344 ± 13µm, also statistically larger than the mean internal diameter of 309 ± 18µm for Baerveldt tubes (P<0.05). The Molteno luminal wall width varied significantly less than the Baerveldt wall, 18% versus 28%, respectively (P<0.05). The tubings’ wall widths variation translated into highly significant off-centered lumens among both brands.

Conclusion

Our findings suggest that there are significant variations among glaucoma implant dimensions between and within the multiple makes and models. The discrepancies among tubal wall thickness and off-centered lumens are undetectable to the naked eye. Importantly, this may result in significant aqueous humor outflow variation following the creation of venting slits secondary to the found irregular luminal diameters and tube wall thicknesses.

## Introduction

Medication-resistant glaucoma is commonly treated with drainage implants among patient populations where other filtration procedures or surgeries may have an otherwise high rate of failure. The implantation of the first glaucoma shunt was developed in the late 1960s by Molteno [1]. Since then, there have been numerous variants with differences among dimensions, materials, and brands [2-4]. However, the commonly used implants are produced by Molteno and Baerveldt, consisting of a plate placed approximated 10mm-13mm posterior to the ocular limbus connected to a silicone-rubber tube inserted into the anterior chamber of the eye [5]. GDD plates may vary in surface area but reportedly have a uniform external tube diameter of approximately 600µm and 300µm internal luminal diameter [6-7]. Proper shunt selection and implantation allow for adequate aqueous drainage, which maintains a decreased yet stable intraocular pressure (IOP) [8].

Although glaucoma drainage devices (GDDs) are considered to have equivalent visual acuity outcomes and disease modifications compared to trabeculectomy procedures, the risk profiles vary over time. In the short term, complications are comparable, including the risk of suprachoroidal hemorrhage, hyphema, choroidal effusion, and endophthalmitis [9]. However, prolonged implantation is associated with an increased incidence of tubal obstruction, conjunctival erosion, and persistent ocular hypotony [9-10]. One of the most common complications found to occur in over a quarter of patients undergoing GDD placement is transient ocular hypotony in the postoperative period [10].

In order to control postoperative ocular pressure, physicians often cut venting slits into the implant’s tubal lumen. This provides aqueous outflow resistance modification to regulate drainage and thus stabilize the intraocular pressure. However, significant variation of IOP has been observed among patients where the venting slit technique and GDD equipment is standardized [11-12]. Our quality-control study was designed to test the hypothesis that this outflow, and thus ocular pressure, variation is due to intrinsic dimensional inconsistencies among glaucoma implant tubing.

## Materials and methods

**Figure 1:**
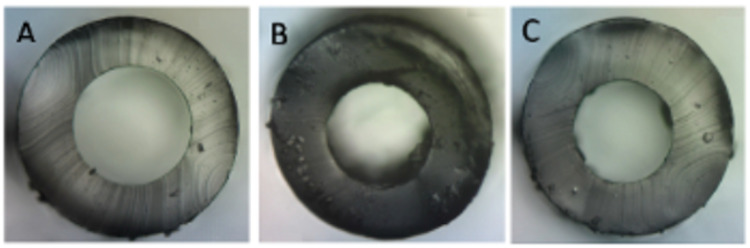
Microscopic images of the glaucoma drainage devices used for analysis A, Molteno-175. B, Molteno-245. C, Baerveldt-250.

Prior to GDD implantation, the excess tubing was trimmed and collected for investigation. We chose the Baerveldt and Molteno GDDs, as these are the two most commonly used brands for glaucoma implants for which venting slits may be performed. Prior to measurement, each sample was carefully cut to a length of 2mm to prevent tubal distortion and flush ends for uniform circumference. The Olympus BX60 microscope (Olympus Corporation, Tokyo, Japan) was used in conjunction with the DinoCapture 2.0 imaging and measurement program (Dunwell Tech, Inc, Torrance, CA) (Figure 1).

**Figure 2:**
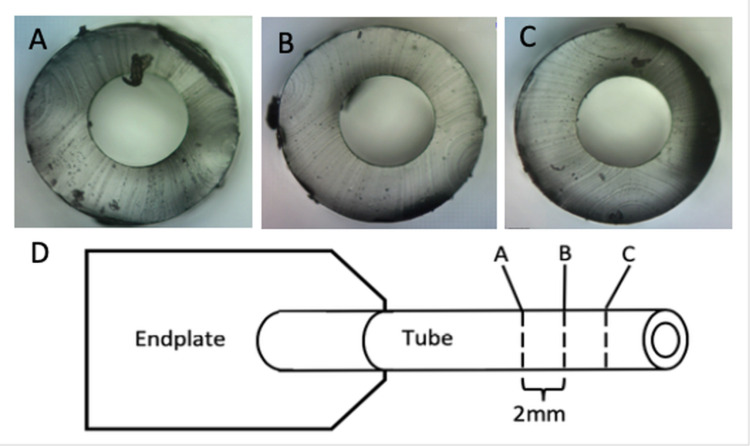
Microscopic images along the longitudinal axis of a Baervedlt-350 separated by 2mm A, Proximal lumen face. B, Middle lumen face. C, Distal lumen face. D, Schematic of the glaucoma drainage device and where the aforementioned samples were taken for measurement.

Each sample’s external tube diameter and internal lumen diameter was recorded to a ± 5µm specificity. The thickest and thinnest portion of each lumen wall was also measured to the same precision. In order to assess the continuity of dimensional variation along the longitudinal axis of each tube, samples were cut into multiple 2mm lengths and then analyzed as stated above (Figure 2). An independent t-test was used for statistical analysis to assess for variation within and between the Molteno and Baerveldt brands.

## Results

A total of 28 tubes were analyzed in this study, consisting of one Molteno-175, three Molteno-185, three Molteno-245, five Baerveldt-250, and 16 Baerveldt-350 for a total of seven Molteno samples and 21 Baerveldt samples. The Molteno brand’s implant had a mean (± SD) external tube diameter of 656 ± 20µm, significantly larger than the Baerveldt tube mean of 620 ± 13µm (P<0.001) (Table 1). The mean internal lumen diameter of the Molteno implants was 344 ± 13 µm, statistically larger than the 309 ± 18µm average lumen of the Baerveldt samples (P<0.001).

**Table 1:** Comparison between the Molteno and Baerveldt glaucoma drainage device external tube diameter, internal lumen diameter, and wall width variation SD, standard deviation.

Glaucoma Drainage Device Dimensions			
	Group	Mean ± SD	P-value
External Tube Diameter, µm	Molteno	656 ± 20	<0.01
	Baerveldt	620 ± 13	
Internal Lumen Diameter, µm	Molteno	344 ± 13	<0.01
	Baerveldt	309 ± 18	
Wall Width Variation, %	Molteno	18 ± 8	<0.05
	Baerveldt	28 ± 12	

The average thickest wall width of the Molteno tube’s lumen wall was 162 ± 10µm compared to the larger Baerveldt’s average thickest wall width of 174 ± 14µm, a difference that was not statistically significant (P=0.38). Additionally, the Molteno sample’s thinnest wall width averaged 138 ± 9µm, insignificantly more than the Baerveldt average of 137 ± 12µm (P=0.18). However, the wall width variation within the individual tubes was significantly larger among the Baerveldt samples (28%) as compared to the Molteno variation of 18% (P<0.05).

## Discussion

Our results support the hypothesis that there is a large variation among the outer intrinsic diameters and inner lumen diameters of GDD tubing, even among the same make and model. Large standard deviations (SD) were found across the implants’ measured dimensions consisting of the external diameter, internal diameter, and luminal wall thickness (Table 1). Notably, the Baerveldt tubes had a larger standard deviation across all dimensions other than external tube diameter when compared to the Molteno brand (P<0.05). Additionally, the Baerveldt brand tubing had a significantly larger variation in lumen wall width, a difference that translates into off-centered lumens, as illustrated by the images (Figure 2).

## Conclusions

These dimensional inconsistencies among glaucoma drainage implants prevent ophthalmologists from making consistent venting slits with GDD tubing. Thus, the tube lumen thickness variability is the likely etiology leading to inconsistent aqueous drainage from venting slits performed by the same technique. Therefore, it is recommended that physicians carefully monitor the titration venting slit flow of each patient’s GDD surgery even if making venting slits with the same technique and instrument. Clinically, the Baerveldt tubing is of a significantly smaller diameter and may make it harder to create a consistently reliable venting slit as compared to Molteno tubing. In addition, both Baerveldt and Molteno had offset lumens producing significantly different wall thickness around the circumference of the tubing. Therefore, unknowingly having a venting slit penetrate the thinnest part of an irregular tube could lead to excess flow and hypotony. Starting with smaller-sided needle punctures may be of use in titrating proper drainage from venting slits. If excess flow is observed after a venting slit is made, a wrapped 9-0 nylon suture to cover the venting area may provide stabilization of outflow and pressure.
